# A phase-1/2 study of adenovirus-p53 transduced dendritic cell vaccine in combination with indoximod in metastatic solid tumors and invasive breast cancer

**DOI:** 10.18632/oncotarget.24118

**Published:** 2018-01-10

**Authors:** Hatem Soliman, Fatema Khambati, Hyo S. Han, Roohi Ismail-Khan, Marilyn M. Bui, Daniel M. Sullivan, Scott Antonia

**Affiliations:** ^1^ Breast Department, H. Lee Moffitt Cancer Center and Research Institute, Tampa, FL, USA; ^2^ Immunology Department, H. Lee Moffitt Cancer Center and Research Institute, Tampa, FL, USA; ^3^ Anatomic Pathology Department, H. Lee Moffitt Cancer Center and Research Institute, Tampa, FL, USA; ^4^ Malignant Hematology Department, H. Lee Moffitt Cancer Center and Research Institute, Tampa, FL, USA; ^5^ Thoracic Oncology, H. Lee Moffitt Cancer Center and Research Institute, Tampa, FL, USA

**Keywords:** indoximod, adenovirus-p53 transduced dendritic cell vaccine, cancer vaccine, immunotherapy, indoleamine 2,3-dioxygenase

## Abstract

**Background:**

Indoleamine 2, 3-dioxygenase is an enzyme that causes immunosuppression in tumors. Indoximod inhibits the indoleamine 2, 3-dioxygenase pathway and enhances immunologic responses to dendritic cell (DC) vaccines preclinically. Adenovirus p53 (Ad.p53) is used to generate DC vaccines against p53. A phase-1/2 trial of indoximod with Ad.p53-DC vaccine was conducted.

**Materials and Methods:**

The phase-1 study combined 7 indoximod dose levels with < 6 Ad.p53-DC vaccinations every 2 weeks. Primary endpoints were maximum-tolerated dose in phase 1 and objective response in phase 2. Flow cytometry measured immune responses.

**Results:**

Thirty-nine patients were treated. In combination with Ad.p53-DC vaccine, the maximum-tolerated dose of indoximod was 1600 mg twice daily. Attributable toxicities were grade 1–2. Best response was stable disease in 4 patients. Immunologic responses were detected in 7 out of 23 evaluable patients. Median progression-free survival was 13.3 weeks (95% confidence interval, 12.97–21.85) and median overall survival was 20.71 weeks (95% confidence interval, 25.75–46.15). Nine out of 22 patients (40%) benefitted from chemotherapy after vaccination. Median overall survival in chemotherapy responders was 69.4 weeks (30.1–122.1).

**Conclusions:**

Indoximod 1600 mg twice daily with Ad.p53-DC was well tolerated. There may have been a chemosensitization effect. Future trials should explore combining this treatment with chemotherapy.

## INTRODUCTION

The main physiologic function of indoximod is as the rate-limiting initial step in the breakdown of the essential amino acid tryptophan into various active metabolites, such as kynurenine and nicotinamide adenine dinucleotide. Indoximod is expressed by various malignant and normal cells in an inducible fashion upon exposure to interferon gamma. The role of indoximod in modulating the immune response was first elucidated by the groundbreaking work of Andrew Mellor and David Munn in 1998, showing it prevented rejection of the fetus in pregnant mice [1]. The authors proposed that indoximod promoted a toleragenic state towards fetal antigens by depleting tryptophan, thereby inactivating the maternal effector T cells in the placenta. Within the tumor tissue, high levels of indoximod expression cause infiltrating effector T cells to arrest in G1, become anergic, and die by apoptosis [2]. The depletion of tryptophan within the tumor causes an increase in the level of uncharged transfer RNAs and activation of the general control nonderepressible 2 kinase-mediated integrated stress response pathway in T cells [3].

The drug indoximod (1-D-methyl-tryptophan) was developed as an orally bioavailable small molecular inhibitor of the indoleamine 2,3 dioxygenase pathway. The preclinical data demonstrated the activity of indoximod in preventing T-cell anergy in tumor-draining lymph nodes, delaying growth of transplanted Lewis lung cancer mouse xenografts, and working synergistically with various chemotherapeutic agents (doxorubicin, cyclophosphamide, paclitaxel) in the regression of autochthonous breast tumors in mouse mammary tumor virus-Neu mice [4, 5]. Finally, Ou et al. demonstrated that indoximod enhanced the anti-tumor efficacy elicited by a dendritic cell (DC)/Lewis lung carcinoma-fusion vaccine administered to Lewis lung cancer mice, by delaying tumor development and inducing stronger splenic cytotoxic T-lymphocyte responses compared to DC/Lewis lung cancer vaccine alone [6]. Phase-1 data available at the time demonstrated indoximod was safe and well tolerated at oral doses of up to 2000mg twice daily. Pharmacokinetic data from this trial also suggested that single doses greater than 1600mg did not result in significantly increased peak serum levels, so this dose was selected as the recommended phase 2 dose. [7]. These data provided a rationale for exploring the safety and activity of indoximod combined with a DC-based vaccine in patients with metastatic disease.

For this trial, we elected to use an autologous DC vaccine produced by infecting the DC cells with the adenoviral vector contusugene ladenovec, containing the wild-type p53 sequence under control of a cytomegalovirus promoter. Many exon 8 to 12 mutations in the p53 sequence result in the abnormal accumulation of dysfunctional protein in cancer cells that can be detected by immunohistochemistry [8, 9]. This excess protein is processed, and p53 epitopes are presented by malignant cells through major histocompatibility class-1 receptors, providing an immunologic target for T cells to recognize and activate against. In a phase-2 small-cell lung cancer trial testing the Ad.p53-DC vaccine, 2 patients developed major tumor regressions that were directly attributable to the vaccine. Also, p53-specific immune responses were detected in 54% of all vaccinated patients. Objective clinical responses to Ad.p53-DC vaccine alone were < 5%, but there was a suggestion of an enhanced response to salvage paclitaxel in immunologic responders [10]. We hypothesized that the addition of indoximod to the Ad.p53-DC vaccine may substantially improve p53-specific immune responses and hence clinical responses. Following approval by the National Cancer Institute’s Cancer Therapeutics Evaluation Program, we launched a phase-1 trial, combining indoximod with Ad.p53-DC vaccine in metastatic breast cancer. The primary endpoint was to identify the maximum-tolerated dose, which would become the dose recommended for phase 2. Phase 2 was a single-arm Simon 2-stage trial in metastatic breast cancer, looking at objective response rates.

## RESULTS

### Patient population

Patient demographics are outlined in Table 1. The majority of patients enrolled were white females with breast cancer, and almost all the patients were pretreated for metastatic disease (at a median of 2 lines of therapy) prior to study enrollment.

**Table 1:** Characteristics of enrolled study population

Median age, y (range)	57 (33–80)
Gender, No.	
Female	40
Male	4
Race, No.	
White	39
African American	5
Tumor types, No.	
Breast	34
Colon	4
Gastric	2
Lung	1
Tongue	1
Ovarian	1
Chondrosarcoma	1
ECOG Performance Status	
0	27
1	1
Median lines prior therapy, No. (range)	2 (0–6)

Abbreviation: ECOG, Eastern Cooperative Oncology Group.

### Patient flow

**Figure 1:**
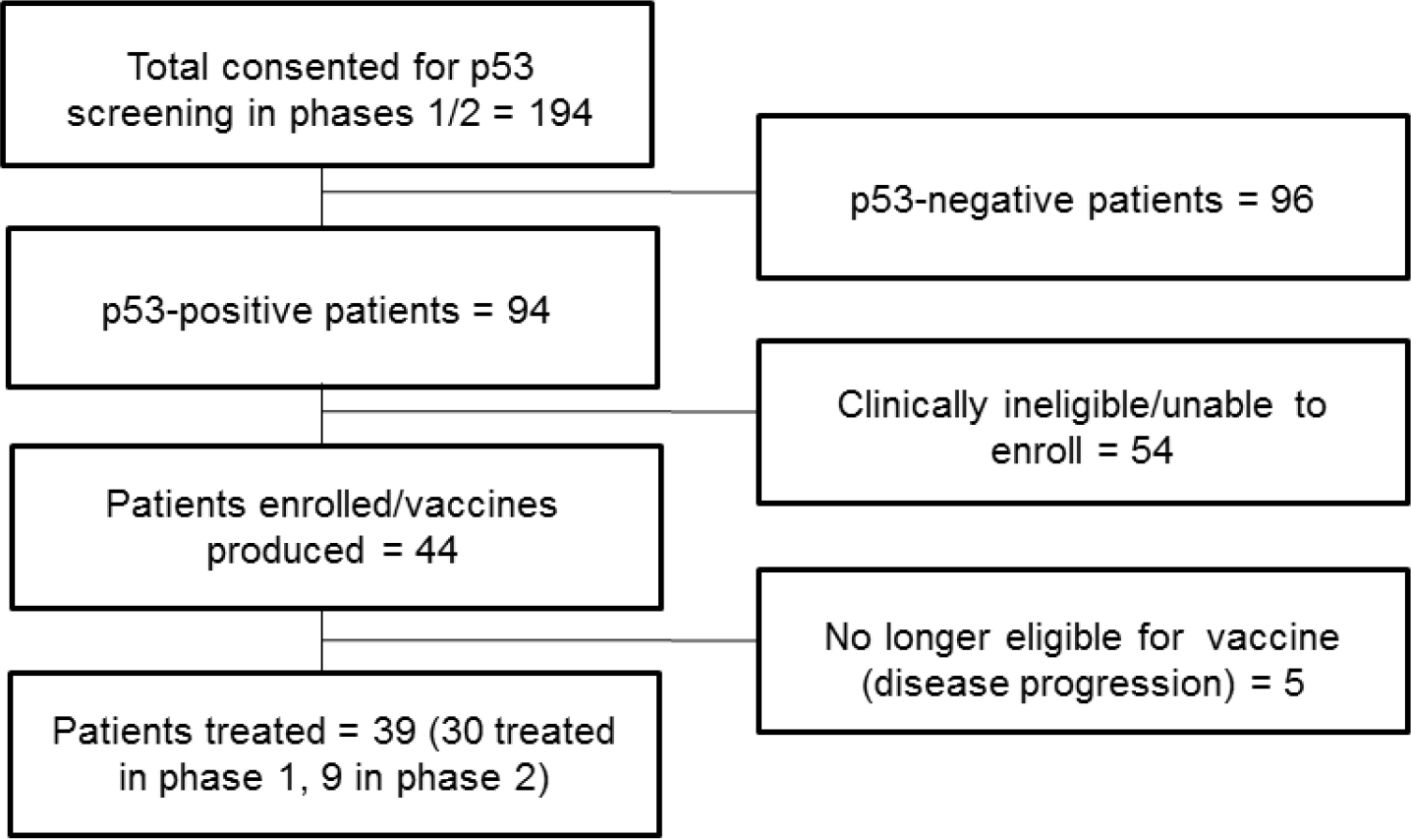
Patient flow diagram

One hundred ninety-four patients consented to screen for the study. Of those patients, 96 failed prescreening because they were p53-negative by immunohistochemistry, and 54 were clinically ineligible for other reasons. This left 44 patients who were enrolled, with 5 (2 in phase 1 and 3 in phase 2) patients unable to get study treatment due to symptomatic progression of disease prior to the initiation of study treatment. The total number of patients who received at least 1 vaccine and 1 dose of indoximod was 39. Thirty of the patients were treated in phase 1, and 9 breast cancer patients were treated in phase 2. Enrollment took place from December, 2009 to February, 2014. Patient flow is depicted in Figure 1.

### Adverse events

Table 2 lists the frequency of any grade 3, 4, or 5 toxicities that occurred. Most, with the exception of anemia, nausea, constipation, and lymphopenia, were not attributed to study treatment. These toxicities are considered expected toxicities for indoximod, based on the results of prior phase-1 studies [7]. The grade 5 dyspnea was a respiratory failure event, which was due to disease progression. The most common grade 1 to 2 adverse events (occurring more than 20% of patients) possibly related to study therapy were fatigue, anemia, transient lymphopenia, nausea, and anorexia. None of the toxicities required treatment discontinuation, and the study treatment was well tolerated overall.

**Table 2:** Grade 3 to 5 adverse events, regardless of attribution

Adverse Event	Grade 3, No.	Grade 4, No.	Grade 5, No.
Anemia	3		
Alkaline Phosphatase	3		
Nausea	1		
Transaminase increase	1	1	
Constipation	1		
Lymphopenia	1		
Back Pain	2		
Cough	1		
Dyspnea	1		1
Ileus	1		
Muscle weakness	1		
Hypoalbuminemia	1		
Hyponatremia	2		
Neutropenia		1	
Abdominal Pain	1		
Hypotension	1		
Bilirubin increase	1		
Platelet decrease	1		
Skin infection	2		
Febrile neutropenia	1		

### Dose-limiting toxicities

No dose-limiting toxicities were encountered during the study, and we eventually reached the maximum-recommended phase-2 dose for indoximod of 1600 mg, administered orally twice a day, in combination with the Ad.p53-DC vaccine.

### Response rate

The best observed responses, as measured by Response Evaluation Criteria in Solid Tumors, were 4 patients with stable disease at week 7. No objective responses were observed during the vaccination treatment period in phase 1- or 2-enrolled patients. Of the 39 treated patients on study, 22 patients were able to go onto at least 2 cycles of subsequent post vaccination therapy and be evaluated for response by their treating physicians. The remainder experienced significant declines due to disease progression and were enrolled in palliative care. The regimens included gemcitabine, carboplatinum/gemcitabine, eribulin, and navelbine. Nine of the 22 patients (40%) had either stable disease or better on imaging (1 complete response, 7 partial responses, 1 stable disease). The patient who had a complete resolution of her lesions as visualized on positron emission tomography/computed tomography occurred following fifth-line carboplatinum/gemcitabine therapy.

### Outcomes

The median progression-free survival for the enrolled study population was 13.3 weeks (95% CI, 12.97–21.85) and median overall survival was 20.71 weeks (95% CI, 25.75–46.15). We did not detect any statistically significant difference in progression-free survival or overall survival between the 7 immunologic responders and the 16 nonresponding patients (data not shown). In the 9 patients who attained a response to salvage chemotherapy following study treatment, the median overall survival was 69.4 weeks (95% CI, 30.1–122.1).

### Immune response data

Of the total study population, only 23 patients yielded sufficient viable cryopreserved PBMCs at both baseline and 3 weeks post vaccination for the immune analysis to be completed. Seven of 23 patients (30%) had > 10% increase in the proportion of CD8^+^ interferon gamma-positive cells compared to their baseline measurements. Six of the 23 patients also demonstrated a > 10% increase in CD8^+^CD69^+^ cells at week 3 compared to baseline. With regards to CD4^+^ interleukin 2-positive helper T cells, 4 out of 23 (17%) had a > 10% increase from baseline at 3 weeks. Of these 4 patients with elevations in helper T cells, 3 also had > 10% increases in their CD8+ T cells demonstrating an overlap between the two immune response groups.

**Figure 2:**
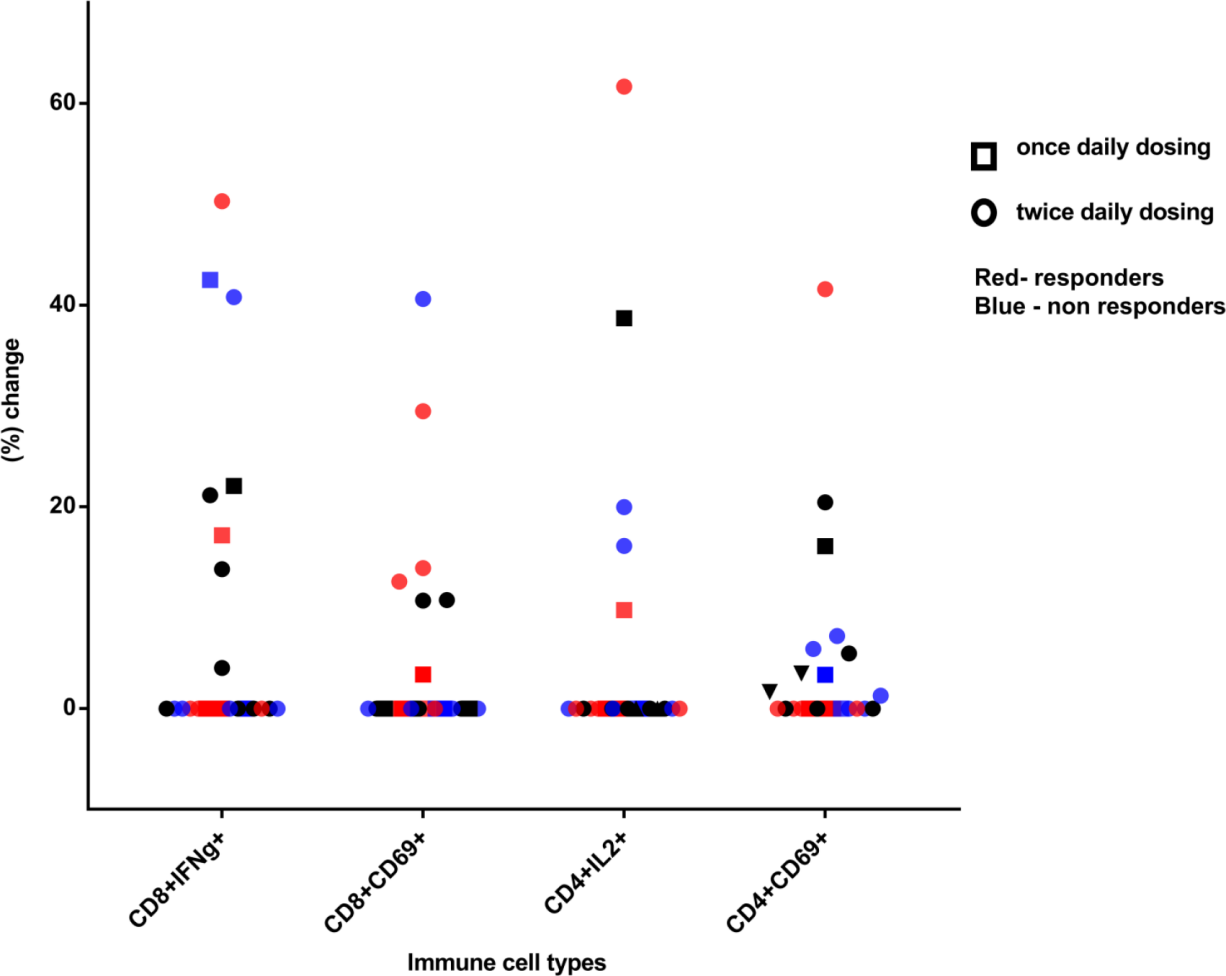
Percentage increase in p53-reactive CD4 and CD8 cells at week 3 post vaccination, compared to baseline Square symbols represent patients enrolled at a once-daily indoximod dose level and circles represent patients enrolled at a twice-daily dose level. Blue shapes represent nonresponders and red shapes represent responders to subsequent chemotherapy. Black shapes represent patients who did not get therapy post vaccine or whose response is unknown.

An analysis looking at the proportion of patients at the higher dose levels of indoximod (800 mg orally twice daily and higher) versus the lower dose levels suggested that a larger proportion of the responsive patients were treated at the higher dose levels (4/7 CD8^+^ interferon gamma positive; 6/6 CD8^+^CD69^+^; 3/4 CD4^+^ interleukin 2 positive; 3/3 CD4^+^CD69^+^). With regards to post study treatment response, it appears that 5 out of the 9 patients with stable disease or better had a > 10% increase in their activated CD8^+^ cell counts (by interferon gamma or CD69) at 3 weeks (Figure 2).

## DISCUSSION

The combination of Ad.p53-DC vaccine and indoximod was safe and well tolerated up to a maximum dose of 1600 mg orally twice daily, with no unexpected adverse events. Monotherapy Ad.p53-DC vaccine trials in metastatic patients did not demonstrate significant objective responses, and the combination of vaccine with indoximod did not increase the objective response rate to our prespecified threshold of 20%. Indoximod also demonstrated limited single-agent activity in phase-1 trials [7], but there does appear to have been some promising activity in recently reported phase-2 studies that combined indoximod with checkpoint therapy [11]. There may be a role for a DC vaccine such as Ad.p53-DC added to indoximod-positive programmed cell death-1 inhibitors to further boost T-cell responses against p53-mutated tumors.

The immune-response data appear to further support the immunogenicity of the Ad.p53-DC vaccine plus indoximod, with responses detected in 30% of treated patients. There were some issues encountered with our patient population that posed some challenges to performing a complete immune profiling on all treated patients. Many of our patients’ samples had poor viability post thaw (limiting the use of other immune-response assessment methods such as Enzyme-Linked ImmunoSpot assay) and limited reactivity to our positive controls. We attribute this to the fact that many patients were enrolled 3 weeks after finishing their prior chemotherapy treatments. Also, the widespread metastatic burden of disease in some of these patients contributed to overall immune dysfunction, further impairing responses to the vaccine. Integrating an autologous DC vaccine into the treatment of metastatic breast cancer patients can be challenging, due to a limited window of opportunity before disease progression becomes excessively symptomatic without systemic therapy. Other settings that allow for longer vaccination schedules with less immune suppression, such as minimal residual disease states in high-risk patients, may provide better patient populations for studying the interaction between indoximod and Ad.p53-DC vaccine. While a greater number of patients at the higher dose levels of indoximod responded to the Ad.p53-DC vaccine, we cannot conclusively demonstrate a dose-response relationship due to our limited sample size and study design.

The ability to sensitize tumors to subsequent systemic chemotherapy using nontoxic vaccines could provide significant clinical benefits to patients and improve quality of life. The follow-up of post vaccination treatment outcomes in our pretreated study population did show that a significant proportion of patients with p53 dysfunctional tumors were able to respond to salvage chemotherapy following vaccination. However, limitations in our study prevent us from attributing the observed responses to any effect by the study treatment. A randomized study with and without Ad.p53-DC with or without indoximod using a standardized post vaccination chemotherapy regimen in a uniform population would be required.

Recent developments in cancer immunotherapy have led to multiple approvals for checkpoint therapies in solid tumors. These modalities can provide more durable tumor control than traditional therapies but only achieve this in a minority of patients. Combination strategies either targeting other immunosuppressive pathways or using stimulatory agents to energize an antitumor response are required to expand the clinical benefit of checkpoint therapy to more patients with metastatic cancer. The fact that the p53 protein is one of the most commonly mutated proteins in cancer makes it an attractive therapeutic target. Immune evasion from a p53-directed immune response may actually eradicate more resistant clones, leaving more sensitive p53 wild-type subclones afterward that can be killed by subsequent treatments. There is a pending phase-2 ovarian cancer trial combining the programmed cell death-1 inhibitor pembrolizumab with a p53-expressing Ankara virus vaccine (NCT03113487). This trial, and hopefully other future studies, will hold the keys to improving outcomes across a wide range of tumor types harboring p53 mutations.

## MATERIALS AND METHODS

### Patient eligibility

Patients with advanced solid tumors were eligible for phase 1 of our study; those with estrogen receptor-positive/negative HER2-negative metastatic breast cancer with measurable disease were eligible for phase 2. Other eligibility criteria included > 5% cells staining positive using p53 immunohistochemical clinical diagnostic assay, age > 18 years, life expectancy > 4 months, Eastern Cooperative Oncology Group performance status 0 to 2, and adequate organ/marrow function. Patients were excluded if they met any of the following criteria: *1*) chemotherapy/radiotherapy within the past 3 weeks, *2*) untreated brain metastases, *3*) uncontrolled concurrent major illness, *4*) current use or previous allergic reaction to L-tryptophan, *5*) active autoimmune disease or chronic inflammatory condition requiring the use of steroids or systemic immunosuppressants, *6*) pregnancy, *7*) AIDS/ HIV infection, or *8*) history of gastrointestinal disease causing malabsorption/obstruction.

### Study design

The protocol was approved by the Food and Drug Administration and the National Cancer Institute’s Cancer Therapeutics Evaluation Program and was conducted in accordance with all federal and institutional guidelines. All patients provided written informed consent under a University of South Florida Institutional Review Board-approved protocol, prior to the initiation of any study procedures. The phase-1 trial (NCT01042535) used a 3 + 3 design with the dosing of Ad.p53-DC vaccine fixed (1-5 x 10^6^ cells/vaccination) and indoximod increased across the following dose levels: 100 mg, 200 mg, 400 mg, 600 mg, and 800 mg once daily and 800 mg, 1200 mg, and 1600 mg twice daily. Six patients were enrolled onto once- and twice-daily 800 mg dose levels, due to the addition of indoximod dose-level amendments during accrual (and not because of dose-limiting toxicities requiring dose expansion). Additional patients were enrolled onto these 2 dose levels while the higher-dose amendments were processed and approved. Dose-limiting toxicity was considered reached when patients experienced grade 3 or higher toxicities, toxicities deemed unacceptable in the opinion of the investigator, or grade 2 or higher non-dermatologic autoimmune events attributed to the study treatment within the first 6 weeks of therapy.

The primary endpoint of the phase-1 trial was to determine the maximum-tolerated dose of indoximod in combination with Ad.p53-DC vaccine. Safety was described using Common Terminology Criteria for Adverse Events version 4.0. The primary endpoint for the single-arm Simon 2-stage phase-2 trial was objective response rates by Response Evaluation Criteria in Solid Tumors version 1.1. Secondary endpoints included the overall objective response rate in phase 1, p53 immune-response flow cytometry correlatives, progression-free survival, and post vaccination therapy response rates in all treated patients.

Indoximod (50 mg and 200 mg hard gelatin capsules) was supplied by the National Cancer Institute’s Pharmacy Branch and NewLink Genetics. It was administered orally twice daily on an empty stomach for 21-day continuous cycles. Patients took indoximod throughout the entire vaccination series and as monotherapy thereafter, if they continued to demonstrate clinical benefit.

The autologous DC vaccine was generated using contusugene ladenovec, a replication-impaired serotype 5 adenoviral vector, containing the wild-type p53 gene under control of a cytomegalovirus promoter (Ad.p53). Ad.p53-DC vaccine was produced in the H. Lee Moffitt Cancer Center and Research Institute Cellular Therapy Core using 1 to 2 blood volumes leukapheresis product that was obtained through Cobe Spectra hemapheresis units. All patients were tested prior to pheresis for human immunodeficiency virus, hepatitis B/C, human T lymphotrophic virus I/II, and syphilis. The cell product underwent an automated Ficoll separation and was stored at –96°C in a 150 mL volume of plasmalyte-A/20% autologous plasma solution with 10% dimethyl sulfoxide. The cells were thawed approximately 1 week prior to vaccination, washed in Cell Genix DC medium, and then adherent DC cells were incubated in tissue culture flasks and activated with 100 ng/mL granulocyte-macrophage colony-stimulating factor and 50 ng/mL interleukin 4 over 4 days. Following activation, the DCs were infected with Ad.p53 at a multiplicity of infection of 15 000 particles per cell and incubated for 48 hours. The vaccine underwent all required quality checks prior to its release for use, including for viability, microbiological contamination, endotoxin, and > 10% positivity for p53-positive DCs by flow cytometry. The final volume (1mL, 1–5 × 10^6^ p53^+^ DC cells) of Ad.p53-DC vaccine was administered intradermally as 4 × 0.25 mL doses distributed over the right and left axillary and inguinal lymphatic basins once every 2 weeks over a 6-week period. If patients had minor progression or better at their first radiologic evaluation post treatment, they could undergo another pheresis to receive additional vaccinations to be administered once every 3 weeks over a 9-week period.

### Safety evaluations

Complete blood counts and metabolic panels were obtained at baseline and every 3 weeks during treatment. Pituitary function tests (thyroid-stimulating hormone, free thyroxine, luteinizing hormone, follicular-stimulating hormone, and adrenocortical hormone) were obtained at baseline and every 5 weeks on study. Patients underwent complete physicals and adverse event evaluations every other week through the first 7 weeks of treatment and every 3 weeks thereafter or as clinically indicated.

### Response evaluation

The overall response rate to the study vaccine was determined via the criteria described by the Response Evaluation Criteria in Solid Tumors 1.1 guidelines. Baseline scans were conducted in the 28-day period before therapy started. Patients were then rescanned on weeks 7, 16, and every 8 weeks thereafter. The best overall response achieved during study therapy was recorded for each patient. The duration of overall response was measured from the time criteria were met for complete or partial response until the first date that recurrent or progressive disease was documented. In patients exhibiting response or disease stabilization, treatment was continued until 1) disease progression, 2) intercurrent illness that prevented further treatment, 3) unacceptable adverse events despite appropriate supportive care, or 4) patient withdrawal from trial. Follow-up scans to determine clinical benefit from salvage chemotherapy off study were interpreted, based on a clinical assessment of benefit by a radiologist.

### Immunohistochemical p53 assay

Anti-p53 (Bp53-11) primary antibody by Ventana Medical System, Inc. (Tucson, AZ) was tested on formalin-fixed and paraffin-embedded breast cancer tissue using citrate buffer (pH6.5) antigen retrieval method and Ventana Benchmark Automated Staining System. Tumors that exhibited > 5% of nuclear immunoreactivity of moderate to strong intensity to p53 were deemed positive.

### Immune response assays

Patient peripheral blood mononuclear cells (PBMCs) were isolated from 15 mL of whole blood at baseline and week 3, using Ficoll Hypaque (GE Healthcare) cryopreserved in 10% dimethyl sulfoxide and stored in liquid nitrogen. Peptide pools for human p53 and cytomegalovirus/Epstein Barr virus/influenza (JPT Innovative) were used at a concentration of 1 µg/mL, as per manufacturer’s instructions. Cryopreserved patient PBMCs (baseline and 3-week post vaccination samples) were thawed and plated in 96 flat-bottom plates at a density of 5 x 10^5^ cells per well in Roswell Park Memorial Institute medium containing 10% fetal bovine serum and 1% penicillin/streptomycin and allowed to rest overnight. The following day, the samples were stimulated with cytomegalovirus/Epstein Barr virus/influenza-positive control peptide (duplicate wells of baseline sample/patient) and p53 peptide (baseline and follow-up samples/patient) at a concentration of 1 µg/mL in Roswell Park Memorial Institute medium and incubated for 5 days at 37°C in 5% carbon dioxide. On day 6, the cells were treated with 5 ng/mL phorbol 12-myristate 13-acetate, and 500 ng/mL ionomycin for 4 hours in the presence of 0.67 µL/mL of Golgi stop solution (BD Biosciences) for intracellular cytokine staining. All the samples were set up in duplicates. Optimization experiments were carried out on normal buffy coats for antibody dilutions and fluorescence-minus-1 experiments were run to generate positive/negative flow gates for the cell populations. Following treatment with phorbol 12-myristate 13-acetate and ionomycin, the PBMCs were washed with 1X phosphate-buffered saline (PBS) and stained with live/dead fixable aqua dye (Biolegend) by incubating them for 20 mins at room temperature. Following washes with 5% bovine serum albumin to remove unbound dye, the cells were stained with brilliant ultraviolet 395-conjugated CD3 (5 µL), phycoerythrin-conjugated CD4 (5 µL), brilliant ultraviolet 737-conjugated CD8 (3 µL), and brilliant violet 421-conjugated CD69 surface antibodies (early-activation markers on lymphocytes) by incubating at 4°C for 1 hour. All antibodies were purchased from BD Biosciences. Following staining, the cells were washed with 1X PBS and fixed for 15 mins at 4°C, as per manufacturer’s protocol, using a fixation permeabilization kit from BD Biosciences. Cells were washed twice with 1X PBS free of fixative and once with permeabilization buffer. Following centrifugation, the cells were re-suspended in 50 *µL* of permeabilization buffer and stained for intracellular cytokines (interferon gamma 5 *µL* and IL-2 5 *µL*) (BD Biosciences) by incubating at 4°C for 1 hour. Following incubation, cells were washed with 1X PBS, finally re-suspended in 400 µL of 1X PBS, and acquired using a BD LSR II cytometer by gating on 10 000 live CD3 cells for each sample. Analysis was performed using FlowJo version 10. The threshold for a positive response to vaccine by week 3 was an increase of > 10% p53 peptide-reactive CD8-positive interferon gamma-positive cells, as compared to each patient’s baseline level.

### Statistics

Descriptive statistics were used for the tabulation of adverse events by frequency and grade, using Common Terminology Criteria for Adverse Events 4.0. Patients who received any dose of the study treatment were evaluable for safety. Median progression-free survival and overall survival were generated with 95% confidence intervals (CIs) for the entire study population, using column statistics analysis in Prism Graphpad 7.03. Log-rank analysis, using Kaplan Meier curves, was performed on the survival outcomes, comparing immune responders with nonresponders. Statistical analysis for the phase-2 optimal Simon 2-stage portion of the study was designed assuming the null hypothesis to be a response rate of 5% for the Ad.p53-DC vaccine alone. The sample size of 37 patients (12 in the first stage, with 1 response required to proceed to the second stage) had 90% power to detect a true response rate of 20% with a 1-sided significance level of .09.

## Abbreviations

Ad.p53-DC: Adenovirus-p53 transduced dendritic cell vaccine
CI: Confidence interval
DC: Dendritic cell
PBS: Phosphate-buffered saline
PBMC: Peripheral blood mononuclear cells
